# How formal caregiver’s BPSD knowledge influences positive aspects of caregiving: the mediating role of attitude and the moderating role of self-efficacy

**DOI:** 10.1186/s12877-022-03417-5

**Published:** 2022-09-05

**Authors:** Rui Hu, Bingbing Lai, Wenhao Ma, Yuan Zhang, Yujiao Deng, Lianqi Liu, Zeping Lv, Chetwyn Chan, Fan Zhang, Qian Tao

**Affiliations:** 1 Department of Public Health and Preventive Medicine, School of Medicine, Jinan University, Guangzhou, China; 2 Division of Medical Psychology and Behavior Science, School of Basic Medicine, Guangdong Province, Jinan University, Guangzhou, 510632 China; 3 Home for the Aged Guangzhou, Guangzhou, China; 4 Department of Rehabilitation, Psychiatric Hospital of Guangzhou Civil Affairs Bureau, Guangzhou, China; 5 Beijing Key Laboratory of Rehabilitation Technical Aids for Old-Age Disability, National Research Center for Rehabilitation Technical Aids, Beijing, China; 6 Department of Psychology, the Education University of Hong Kong, Hong Kong, Hong Kong; 7 Center for Brain Science and Brain-Inspired Intelligence, Guangdong-Hong Kong-Macao Greater Bay Area, Guangzhou, China; 8 Neuroscience and Neurorehabilitation Institute, University of Health and Rehabilitation Science, Qingdao, China

**Keywords:** BPSD knowledge, Positive aspects of caregiving, Dementia attitude, Self-efficacy, Formal caregivers

## Abstract

**Background:**

The current study investigated the relationship between behavioural and psychological symptoms of dementia (BPSD) knowledge and positive aspects of caregiving (PAC), in addition, how caregiving attitude and self-efficacy mediate or moderate this relationship.

**Methods:**

Two hundred twenty-nine formal caregivers (51males and 178females) who has worked in nursing homes for more than a month were recruited.With a cross-sectional, face-to-face survey, structural questionnaires were implemented to evaluate formal caregiver’s BPSD knowledge, attitude, self-efficacy and PAC.A 13-item self-developed questionnaire was used to assess caregiver’s BPSD knowledge about disease characteristics, care and risks, and treatment needs. Dementia attitude, self-efficacy and positive aspects of caregiving were measured by dementia attitude scale, the General self-efficacy scale, and Chinese version of positive aspects of caregiving respectively. Model 5 in the PROCESS micro was employed in order to verify the mediating effect of attitude and the moderating effect of self-efficacy on the relationship between BPSD knowledge and PAC.

**Results:**

The results showed that greater BPSD knowledge was associated with increased PAC, and this relationship was fully mediated by increased friendly attitude toward people with dementia. Moreover, direct effect was moderated by self-efficacy, and that only among those with high self-efficacy, the direct effect of BPSD knowledge was found on promoting PAC.

**Conclusions:**

By elucidating the knowledge-attitude-practice pathway in handling patient’s BPSD, the current study extends existing literature and provides insights for developing psychoeducation programs among formal caregivers.

## Introduction

As a global public health challenge, the prevalence of dementia increases exponentially with age [1]. According to the Global Burden of Disease Study 2016 [2], there were 43.8 million people living with dementia worldwide, and this number is projected to be doubled in 2030 and tripled in 2050 [3]. As a growing number of older adults suffered with dementia have resided in nursing homes, how to ensure the quality of care for people with dementia (PwD) has become a pivotal concern of the public. Taking care of PwD during the pandemic might be more challenging, due to their greater vulnerabilities to the SARS-CoV-2 virus. The high risk of infection also casts a negative impact on healthcare worker’s health and well-being mentally and physically [4]. Given such a background, it is critical to address the protective factors that promote caregivers’ positive reactions.

Caregiver’s experiences and reaction is a response to a myriad of physical, psychological, emotional, social and financial stressors, and the majority of existing literature has focused on caregiving burden. However, caregiving burden is not the only outcome of caregiving, and increased research has found that in the adaptation process of caregiving, both positive and negative experiences could co-exist. For example, recent evidence showed that taking care of PwD can lead to unexpected benefits, such as recognizing one’s own strength of resilience, patience, and fortitude, as well as gratification of interpersonal connections [5]. Overlooking the positive aspects may lead to biased understanding about dementia caregiving experiences. The positive aspects of caregiving (PAC) were characterized as uplift, gratification, reward, growth, and satisfaction among the caregivers [6]. Prior research has found that there are three groups of factors that would determine nursing home staffs’ satisfaction about work: staff characteristics, patient’s characteristics, and contextual factors [7]. Care staffs with more experiences of working [8], greater knowledge about PwDs [9], and more positive attitude [10] usually are more satisfied with caregiving. Adjusting perspective, attitude and behaviors to make meaning is also associated with more PAC during the pandemic [11]. A recent review has suggested that the emergence of PAC could be promoted by the individual factors such as greater self-efficacy [12]. Yu et al. proposed an integrated theoretical model predicting PAC [13], which called for more attention on how to generate the enriching and meaningful aspects of caregiving, and caregiver factors such as knowledge and management skills, self-efficacy should be considered.

### Factors predicting PAC

Existing literature has identified that factors such as knowledge, attitude, and self-efficacy could affect caregiving experiences such as burden or PAC [14, 15]. One of the most challenging factors that influences caregiving experiences is handling PwD’s behavioral and psychological symptoms related with dementia (BPSD), and how increasing knowledge helps with handling BPSD is not clear. In a recent review [16] on formal caregiver’s burden, PwD’s need-driven and emotional behaviors both contribute to greater caregiver burden [17]. The negative impact of disruptive behaviors affecting others (e.g., aggressions, screaming, and repetitions) on staff’s burden could even exceed that of cognitive symptoms [18]. Nevertheless, recent research has viewed BPSD as responsive or need-driven behaviors that serve as PwD’s means of communication [19], and in-depth understandings and knowledge about BPSD would improve caregiver’s competence of handling behavioral symptoms [20], develop adaptive expectations, and result in more positive caregiving experiences. A recent review on caregiver’s competence of handling BPSD has defined this competence as “the aggregation of knowledge, skills, and attitudes as well as the synergies between them” [20]. The authors proposed a conceptual model including four key attributes: judging, empathizing, adjusting, and reflecting. Among them, judging mainly refers to “understanding BPSD incidents”, which was very similar to the BPSD knowledge in our study, and it is the precondition for the other three. Empathizing refers to the warm attitude and the ability to recognize PwD’s emotions behind BPSD. With empathy, the carers could understand and respond to PwD’s needs in a better way. However, how the four attributes interplay with each other in predicting caregiving outcomes still needs more empirical studies.

### The moderating role of self-efficacy

The majority of the research on dementia knowledge has focused on the overall knowledge, and little evidence could be found on knowledge about BPSD and care staff’s PAC. Previous studies showed that the majority of general practitioners reported lacking self-confidence in managing BPSD [21], and it is not clear whether limited knowledge about BPSD is related with low self-efficacy [22]. Further studies are warranted to clarify the role and pathways of BPSD knowledge in caregiving experiences. In addition, the moderating role of self-efficacy would also be investigated in the current study. Self-efficacy, defined as a cognitive construct to which extent an individual believes he or she can fully accomplish a specific task when facing various obstacles [23]. As suggested by a systematic review, feeling competent or having higher self-efficacy would generally reduce caregiver burden and promote caregiver’s mental health [24]. In addition to the protective effect, the self-efficacy in controlling upsetting thoughts also have a moderating effect in the negative impacts of PwD’s behavioural symptoms. This moderation effect of self-efficacy was supported by multiple studies [25, 26]. However, it is not clear whether self-efficacy would also moderate the direct effects of BPSD knowledge.

### The present study

To fill the abovementioned research gap, the current study aimed to address the pathways linking BPSD knowledge and PAC with a self-developed measurement on caregiver’s BPSD knowledge. A cross-sectional study was conducted among healthcare workers at nursing home during the pandemic of COVID-19. The research purpose is two-fold: 1) to address the effects of BPSD knowledge on PAC; 2) to test how attitude and self-efficacy play a role in this effect. The findings will provide further evidence for tailoring intervention programs supporting healthcare workers who provide care for PwDs at nursing homes.

**Fig. 1:**
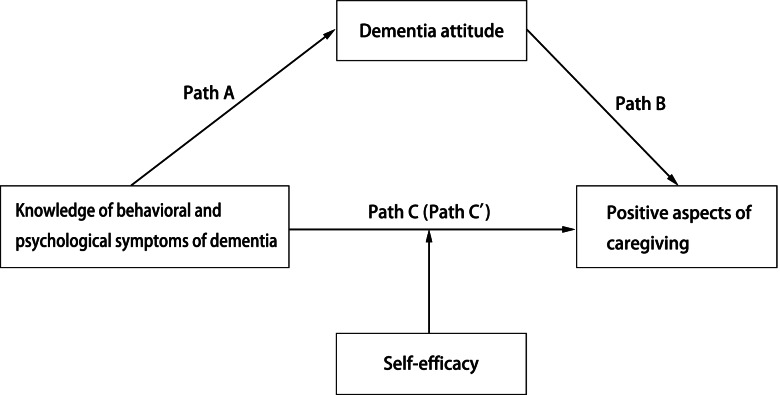
Model hypothesis.The associations between BPSD knowledge and PAC were the total effect (path C), which consisted of a direct effect of BPSD knowledge (path C’) and an indirect effect via attitude toward patient with dementia (path A * path B). Formal caregivers’ self-efficacy was included as a potential moderator in path C’

Based on the findings reported in earlier studies and in view of the existing gap in research literature we propose the two hypotheses (Fig. 1) as below.

Hypothesis 1: Nursing staff with better BPSD knowledge are more likely to report PAC, and this relationship could be at least partially explained by improved attitude toward the PwDs. Hypothesis 2: The positive effects of BPSD knowledge on promoting PAC could be more salient among the nursing staff with strong self-efficacy.

## Methods

### Design and participants

The study has adopted a cross-sectional design, and structural questionnaires were administered. With a convenient sampling method, the study recruited formal caregivers from four nursing homes in Guangzhou and Nanchang, China. Caregivers who met the following criterion are included: 1) over 18 years old; 2) having been working as a formal caregiver for patients with dementia for more than one month; 3) having no difficulties in communicating, understanding and answering the questionnaire. The current study was reviewed and approved by the Ethics Committee of XXX University (Blinded for review), and all participants were asked to provide written consent before initiating the survey.

### Measurements

Formal caregivers were asked to report their demographic information such as gender, age, marital status, education, income. In addition, their care experiences were also evaluated by the questions below “how long you have been providing care for people with dementia”, “how much time do you take care of the patient each day?”, and “does anyone (colleague, family members, etc.) assist you in caring for the patient?”.

Caregivers’ knowledge about BPSD were assessed by the self-developed knowledge scale for the behavioural and psychological symptoms of dementia (KS-BPSD, [39]). KS-BPSD is a 13-item scale included three domains, namely disease characteristics, care and risks, and treatment needs. The total score was obtained by adding up the number of correct responses. The scale showed good test–retest reliability (correlation coefficient = 0.89, *p* < 0.001), and the Cronbach’ alpha was 0.81. Score of each item was significantly correlated with its corresponding subscale (*P* < 0.01, *r* > 0.4). Moreover, *r* between the item and the subscale was higher than that between the item and other subscales. The results showed that KS-BPSD have good convergent and discriminant validity.

Dementia attitude was measured by the 20-item dementia attitude scale (DAS, [27]. DAS used a 7-point Likert scale to measure the affective, behavioural and cognitive components of the attitudes towards people with dementia. There are two factors in DAS, dementia knowledge and social comfort. Total score was obtained by adding up all the item scores, and higher score indicated more positive attitude. The scale showed good reliability in our sample, and the Cronbach alpha was 0.85. After calculating item-subscale correlation, the convergent and discriminant validity is acceptable.

Formal caregivers’ self-efficacy was assessed by the General self-efficacy scale [28]. As a 10-item scale, it used a 4-point Likert scale, ranging from 1 “strongly disagree” to 4 “strongly agree”. Total score was obtained, and higher score reflects better perceived capacity to cope with daily hassles and to adapt to stressful experiences. GSES showed a good reliability in our sample with a Cronbach alpha of 0.92. The score of each item was significantly correlated with the total score (*P* < 0.01, *r* > 0.4), which show a good convergent validity in our sample.

The Chinese version of positive aspects of caregiving was used to measure the positive experiences of caregiving [29]. There are 9 items from two subscales, i.e., affirming self, and enriching life. With a 5-point Likert scale, participants choose from 0 (strongly disagree) to 4 (strongly agree) to address their positive feelings about providing care for people with dementia. PAC showed good validity and reliability, and the Cronbach alpha is 0.91. After calculating item-subscale correlation, the convergent and discriminant validity is good.

### Statistical analysis

Using the approach of Baron and Kenny [30], a mediation model was conducted to analyse the direct and indirect effect of BPSD knowledge on PAC. The associations between BPSD knowledge and PAC were the total effect (path C), which consisted of a direct effect of BPSD knowledge (path C’) and an indirect effect via attitude toward patient with dementia. The association between BPSD knowledge and attitude was path A, and the association from attitude to PAC was path B. Formal caregivers’ self-efficacy was included as a potential moderator in path C’. The hypothesized model was displayed in Fig. 1. The significance of mediating effects was assessed on the basis of the bias-correction bootstrapped 95% confidence intervals, using PROCESS Macro 3.0, SPSS Statistics 24 [31]. Based on the correlation analysis, the model predicting PAC was adjusted for daily care time, training experience, sex, income, years of care.

## Results

### Descriptive results

229 formal caregivers have participated in the study (22.3% male), and the mean age was 43.97 (SD = 10.87). Over 80% participants were married (81.7%), and approximately half of the participants have obtained an educational level above college (47.2%), and 25.3% had an annual income below CNY 30,000 (for details, see Table 1). In addition, 54.6% of the participants have reported a caregiving experience over 3 years, and 64.6% provided daily care longer than 8 h. The majority of the participants (74.7%) reported that there is someone who assists them in caring for the patient, and 80.8% have participated in education programs related to dementia care.

**Table 1:** Demographic Information

Variables	n (%)
Sex	
Males	51(22.3)
Females	178(77.7)
Age	43.97 ± 10.87(M ± SD)
Marital status	
Married	187(81.7)
Other	42(18.3)
Education	
Junior high school and below	72(31.4)
High school/ Technical secondary school	49(21.4)
University/ Junior college	105(45.9)
Postgraduate and above	3(1.3)
Annual income	
< 30,000	58(25.3)
30,000–80,000	87(38.0)
80,000–300,000	71(31.0)
≥ 300,000	13(5.7)
How long you have been caring for people with dementia	
1–6 months	24(10.5)
6–12 months	33(14.4)
1–3 years	47(20.5)
3–10 years	59(25.8)
Over 10 years	66(28.8)
How much time do you take care of the patient each day	
< 2 h	12(5.2)
2–4 h	18(7.9)
4–8 h	51(22.3)
> 8 h	148(64.6)
How many patients are you currently caring for	
1	23(10.0)
2–3	34(14.8)
4–5	40(17.5)
≥ 6	132(57.6)
Does anyone (colleagues, family members, etc.) assist you in caring for the patient	
Yes	171(74.7)
No	58(25.3)
How is the daily living ability (Eating, bathing, urinating, dressing) of the patient you are caring for	
Need help in all daily activities	105(45.9)
Some activities need help	112(48.9)
Take care of himself/herself in all daily activities	12(5.2)
Whether they have participated in education programs related to dementia	
Yes	185(80.8)
No	44(19.2)

### Linear regression predicting PAC

Based on the correlation with PAC, daily care time (B = 1.85, se = 0.70, t = 2.67, *p* < 0.01), training experience (B = 3.71, se = 1.25, t = 2.97, *p* < 0.01), sex (B = 0.49, se = 1.22, t = 0.41, *p* = 0.69, age (B = 0.10, se = 0.07, t = 1.50, *p* = 0.13), income (B = -2.10, se = 0.76, t = -2.79, *p* < 0.01), years of care (B = 0.86, se = 0.40, t = 2.12, *p* = 0.04), were included in the regression model predicting PAC. The results showed that after controlling for the covariates respectively, greater BPSD knowledge was associated with more PAC (B = 0.24, se = 0.08, t = 2.84, *p* < 0.01), suggesting the total effect of BPSD knowledge on PAC was statistically significant.

### Moderated mediation analysis predicting PAC

To test the direct and indirect effects of BPSD knowledge on PAC with self-efficacy as a moderator, PROCESS Macro model 5 was used. As shown in Table 2, after adjusting for the covariates, people with better BPSD knowledge report more positive attitude toward PwD (B = 0.83, se = 0.17, 95%*CI* = [0.49,1.17]), confirming path A of the mediation model. Positive attitude was also found to be related with more PAC (B = 0.15, se = 0.03, 95%*CI* = [0.09,0.21]), confirming pathway B. The mediation analysis showed that the indirect effect of BPSD knowledge via attitude was statistically significant (B = 0.12, se = 0.04, 95%*CI* = [0.06, 0.20]), and the direct effect of BPSD knowledge was not (B = 0.11, se = 0.08, 95%*CI* = [-0.05,0.28]). The results suggested that attitude toward PwD fully mediated the effect of BPSD Knowledge on promoting PAC. When including self-efficacy as a moderator in the direct effect on PAC (pathway C’), the moderated mediation model was statistically significant (F = 17.01, *p* < 0.01), and an interaction effect between BPSD knowledge and self-efficacy was found (B = 0.03, se = 0.01, t = 2.42, *p* = 0.02, See Table 3). Simple effect analysis showed that only among those with high self-efficacy, BPSD knowledge was found to be associated with more PAC (B = 0.24, se = 0.11, 95%*CI* = [0.02,0.45], See Table 4).

**Table 2:** Mediation model

	B	SE	95%CI
Effect of BPSD knowledge on attitude (a)	0.83	0.17	(0.49,1.17)
Effect of attitude on PAC (b)	0.15	0.03	(0.09,0.21)
Direct effect of BPSD knowledge on PAC (c')	0.11	0.08	(-0.05,0.28)
Indirect effect via attitude (a ∗ b)	0.12	0.04	(0.06,0.20)
Total effect (c' + a ∗ b)	0.23	0.08	(0.07,0.40)

*BPSD* Behavioral and Psychological Symptoms of Dementia, *PAC* Positive Aspects of Caregiving, *B* Unstandardized coefficients, *SE* Standard Error, *CI* Confidence Intervals

**Table 3:** Moderated mediation model

	attitude			PAC		
	B	SE	t	B	SE	t
BPSD knowledge	0.83	0.17	4.80^**^	0.05	0.07	0.68
self-efficacy	-	-	-	0.43	0.06	6.89^**^
attitude	-	-	-	0.11	0.03	3.79^**^
BPSD knowledge * self-efficacy	-	-	-	0.03	0.01	2.42^*^
R^2^	0.29			0.44		
F	12.97^**^			17.01^**^		

*BPSD* Behavioral and Psychological Symptoms of Dementia, *PAC* Positive Aspects of Caregiving, *B* Unstandardized coefficients, *SE* Standard Error

^**^p < .01,^*^ *p* < .05

**Table 4:** Conditional direct effects at different levels of self-efficacy

	B	BootSE	95%BootCI
M-1SD	-0.14	0.11	(-0.35, 0.07)
M	0.05	0.07	(-0.10, 0,20)
M + 1SD	0.24	0.11	(0.02, 0.45)

*B* Unstandardized coefficients, *BootSE* Bootstrapped Standard Error, *BootCI* Bootstrapped Confidence Intervals

To sum up, positive attitude has fully mediated the protective effect of BPSD knowledge on PAC, and direct effect was moderated by self-efficacy. Only among those with high self-efficacy, the direct effect of BPSD knowledge was found on promoting PAC.

## Discussion

With a self-developed instrument assessing care staff’s BPSD, the current study has delineated the pathways between BPSD knowledge, attitude, and care staff’s positive experiences of caregiving. The mediation role of attitude and the moderation role of self-efficacy were supported, and such a moderation and mediation model has advanced existing literature on how to promote PAC among nursing home staff.

The pathways between BPSD knowledge, attitude, and PAC provided supporting evidence for the conceptual model of competence in managing BPSD. Meanwhile, it is also in line with the knowledge, attitude, and practice (KAP) model in health. Being commonly adopted in health research, the KAP model involved three steps in caregiving practice: knowledge acquisition, attitude change, and behavioral outcomes [32, 33]. Evidence has shown that more knowledge about PwD’s condition, via increased positive attitude, was associated with better care ability [34], less burden [35], and more perceived gaining according to our findings. The gap in knowledge and attitude of PwD/caregiver is one of the major obstacles in providing high-quality dementia care [36]. By looking at the effects on PAC among nursing home staff, the current study added a new piece to the existing literature on KAP in dementia care research. In our results, the direct effect of BPSD knowledge on PAC was not found. In fact, the relationship between knowledge and PAC has remained inconclusive in existing literature. A recent cross-sectional study has found caregivers who received psychoeducational interventions would have better dementia knowledge, which was associated with greater PAC [37]. However, according to a meta-analysis [38], little evidence could be found on the positive effects of knowledge on PAC after participating the psychoeducational interventions. Therefore, other protective factors or moderators should be considered in the relationship between knowledge and PAC.

In our results, the direct effect between BPSD knowledge and PAC pathway was moderated by self-efficacy, that it only existed among the nursing staff with high self-efficacy. The findings were aligned with the existing literature on dementia care. Nogales-González et al. [25] found that the caregivers with high self-efficacy, they reported lower level of distress related with PwD’s depressive and disruptive behaviours, but not memory problems. Both this study and our findings supported Bandura’s self-efficacy theory, that better perception about one’s capacity of managing a problem is related with better outcomes. Regarding cognitive symptoms such as memory problem, maybe it is more frequent and less likely to be reduced by caregiver’s reaction, and different adaption process was developed. It also suggests that it is more important to educate care staff about the incidence of BPSD with emphasizing that it is more reversible compared with cognitive symptoms, to cultivate their self-efficacy. Otherwise, more knowledge about BPSD may not necessarily promote PAC among the professional carers.

## Conclusions

The current study investigated the direct and indirect effects of nursing staff’s BPSD knowledge on the positive experiences of caregiving. It was found that the positive effects of BPSD knowledge was explained by increased friendly attitude toward PwD, and direct effect was only statistically significant among those with higher self-efficacy. By elucidating the knowledge-attitude-practice pathway in handling patient’s BPSD, the current study extends existing literature and provides insights for developing psychoeducation programs among nursing staff. There are a few limitations that need to be acknowledged. First, since the study was conducted with a face-to-face interview from January 10 to September 16, 2021, a period that is under significant impact of COVID-19, we could only recruit 229 participants from four nursing homes. A larger sample size may be needed to test the generalizability of the moderated mediation model. Second, we only focused on the knowledge about BPSD, and it is hard to answer whether other types of dementia knowledge would have different pathways linking to PAC. Further studies could compare BPSD knowledge with other knowledge to provide more insights for tailoring effective psychoeducation programs. Last, it would be interesting to explore how contextual factors such as the pandemic interplay with nursing staff’s personal factors. Despite the fact that we do not have pre-pandemic data, a post-pandemic data might be collected to allow for longitudinal analysis.

## Data Availability

The datasets analyzed during the current study are available from the corresponding author on reasonable request.

## Abbreviations

BPSD: Behavioural and psychological symptoms of dementia
PAC: Positive aspects of caregiving
PwD: People with dementia
KS-BPSD: Knowledge scale for the behavioural and psychological symptoms of dementia
DAS: Dementia attitude scale
KAP: knowledge, attitude, and practice
